# Dual-Polarization Ku-Band Compact Spaceborne Antenna Based on Dual-Reflectarray Optics †

**DOI:** 10.3390/s18041100

**Published:** 2018-04-05

**Authors:** Carolina Tienda, Jose A. Encinar, Mariano Barba, Manuel Arrebola

**Affiliations:** 1 Airbus Defence and Space, Gunnels Wood Road, Stevenage SG1 2AS, UK; 2 Information, Processing and Telecommunications Center, Universidad Politécnica de Madrid (UPM), 28040 Madrid, Spain; jose.encinar@upm.es (J.A.E.); mbarba@etc.upm.es (M.B.); 3 Department of Electrical Engineering, University of Oviedo, 33203 Gijón, Spain; arrebola@uniovi.es

**Keywords:** dual-reflectarray, contoured beam, Ku-band

## Abstract

This article demonstrated an accurate analysis technique for dual-reflectarray antennas that take into account the angle of incidence of the impinging electric field on the main reflectarray cells. The reflected field on the sub and the main reflectarray surfaces is computed using Method of Moments in the spectral domain and assuming local periodicity. The sub-reflectarray is divided into groups of elements and the field radiated by each group is used to compute the incident and reflected field on the main reflectarray cells. A 50-cm demonstrator in Ku-band that provides European coverage has been designed, manufactured and tested to validate the analysis technique. The measured radiation patterns match the simulations and they fulfill the coverage requirements, achieving a cross-polar discrimination better than 25 dB in the frequency range: 12.975–14.25 GHz.

## 1. Introduction

Synthetic Aperture Radar [1] and long distance communications are two of the most popular and demanding applications for spaceborne antennas that require contoured beams [2,3], multibeam or beam scanning. Reflectarray antennas are a very competitive solution for these applications compared to conventional reflector and phased array antennas. The manufacturing photo-etching technology on multiple layer printed circuit boards, is a very controlled and well-known process. The bandwidth limitations of the printed elements and the differential spatial phase delay [4,5] can be overcome with several techniques like: multi-layer designs [6,7,8] multi-resonant elements, aperture-coupled patches with delay lines [9], or faceted configurations [10].

Dual-reflectarray antennas present some advantages respect to the well-known single offset ones [11], like the reduction of the volume for antenna geometries with a large focal distance. Another advantage is the possibility of using two reflectarray surfaces to increase the functionality of the antenna, or improve its electric characteristics. In the last years, a reflectarray as sub reflector has been combined with large main parabolic reflectors, the high gain characteristics of the reflector are combined with the degree of freedom of a reflectarray subreflector. A sub-reflectarray can improve the behavior of the antenna, either correcting the surface errors of the deployable surface [12], or providing beam scanning within certain angular range [12,13]. When using electronic controlled elements like MEMs [14,15,16], PIN diodes [17,18], varactor diodes [19] or liquid crystal elements [20,21,22] for frequencies higher than 100 GHz.

Antennas implemented with two reflectarrays [13,23,24], one as sub reflector and one as main reflector, provide a high number of degrees of freedom. In a dual-reflectarray antenna, the phase control in both surfaces can be used to improve the performance of the antenna in several ways, depending on the application.

Some of the advantages of using dual offset reflectarray antennas are: They have a high degree of compactness. They are useful to design multibeam antennas with a stable performance in all beams. By implementing the principle of bifocal antennas [25], the sub reflectarray can be used to change from linear to circular polarization or to re-configure the beam, while using the main reflectarray for additional functionalities, or improvements of the antenna performance. Some of the applications of this kind of antennas are Synthetic Aperture Radar [26], radiometric remote-sensing and multibeam antennas for mobile communications.

Reference [24] reported an analysis technique for dual-reflectarray antennas. The reflected coefficient of the elements in both reflectarrays were computed by Spectral-Domain Method of Moments, assuming local periodicity. The reflection coefficient is a matrix that relates the incident and reflected fields of every cell of the main or the sub reflectarrays.

The accurate analysis of a dual-reflectarray antenna is complicated and requires a high computational effort. For every single pair combination of elements of the sub and the main reflectarray, a reflection coefficient matrix must be computed. If NMR and NSR are the number of cells in the main and sub reflectarrays respectively, NMR×NSR would be the number of reflection coefficient matrices that must be computed for an accurate analysis of the antenna.

The incident electric field on every cell of the main reflectarray was approximated by a single plane wave. In the fix incident angle approximation, the reflected coefficient matrices of the main reflectarray are computed NMR times instead of NMR×NSR times as it is done in the full wave analysis. This approximation reduces significantly the computation time. In this approximation, the reflected field on every cell of the main reflectarray is computed only once, assuming the incident angle as the one coming from the center of the sub reflectarray, as the superposition of all the field contributions from every cell of the sub reflectarray. There are some antenna geometries where the reflected field on every cell on the main reflectarray is practically not affected by the incident angle from different sub reflectarray cells. In that cases, the approximation of the fix incident angle is valid, like the compact dual-reflectarray antenna in Ku-band to produce a pencil beam presented in [27]. In that antenna, the elements in the main reflectarray were chosen to be less sensitive to the incident angles, the period was very small (0.4λ × 0.4λ at 15 GHz) and even when having incident angles up to 50° in some elements at the border of the main reflectarray, a good agreement between measurements and simulations was achieved. However, in other antenna configurations, the approximation of the fix angle used in the main reflectarray may not be accurate enough. When using sensitive elements to the incident angle, large antennas or contoured beam antennas. In this case, for Direct Broadcast Satellite (DBS) applications [2,3,28], phase errors may produce non acceptable distortions on the contoured radiation pattern.

A general analysis technique is presented in this paper to provide an accurate model for dual-reflectarray antennas. The incident angles of every element of the sub and main reflectarrays are considered in the analysis, improving significantly the accuracy in the prediction of the radiation pattern. The analysis technique presented in [24] is a particular and simplified case of the general analysis technique presented in this article. Particularly it corresponds to the approximate and less accurate case and cannot be used for a good prediction of contoured beams and crosspolar component of the radiation patterns, which is shown in this paper. A dual-reflectarray technique for contoured beam applications has been implemented. A demonstrator to provide DBS European coverage has been designed, manufactured and tested. The size of the demonstrator was limited to 50 cm to simplify the manufacturing process. The antenna geometry was selected to limit the phase variation on both reflectarray surfaces to 360° and avoid the need of multi-layer phase-delay elements. This paper demonstrates that a dual-reflectarray antenna can produce a contoured beam, emulating a dual offset parabolic reflector of a large F/D (F/D=4). The antenna was manufactured and tested at Universidad Politécnica de Madrid, achieving good agreement between simulations and measurements in the whole designed frequency band (12.975–14.250 GHz).

## 2. Analysis Technique for Dual-Reflectarray Antennas

The proposed analysis technique is used for a dual-reflectarray antenna that consists of a flat sub-reflectarray (SRA) illuminated with a feed horn and a flat main reflectarray (MRA). Both reflectarray surfaces are based on phasing elements of any type implemented with one or more layers of printed patches arranged in a regular lattice. The technique consists of four steps: first, the computation of the incident field on the SRA coming from the primary feed horn. Second, the analysis of the SRA using SD-MoM, assuming local periodicity, and taking into account the angle of incidence of the electric field coming from the feed-horn. Third, the SRA is divided into groups of elements (sub-arrays), and the elements of the MRA are analyzed considering the incident angle and the field contribution coming from each of the SRA groups, using the same technique as for the SRA: SD-MoM and local periodicity. The last step consists on the computation of the radiation pattern, through a 2D-FFT of the reflected field on the MRA. This is a very general approach since it allows adjusting the level of accuracy in the analysis considering a higher or lower number of groups in the SRA. The election of the number of groups is done depending on the antenna geometry demand, based on the maximum angles of incidence and the periodicity. The reflection coefficients of every cell in the MRA (m,n), due to the plane wave coming from a cell of the SRA (p,q), are giving by a matrix $R_{\left(p,q\right)}\left(m,n\right)$. The coefficients of this matrix depend on which defines the maximum angles of incidence on the MRA, on the period and type of cells used in the MRA. The CPU time computation of the $R_{\left(p,q\right)}\left(m,n\right)$ matrix is very high since all combinations of cells (m,n)-(p,q) must be calculated.

For some antenna geometries where the MRA is in the Fresnel zone of the SRA, the incident angles of the field contributions from different cells are very similar. These incident angles can be approximated as done in [24] where $R_{\left(p,q\right)}\left(m,n\right)\approx R\left(m,n\right)$, being R(m,n) the reflection matrix corresponding to the incident field coming from the center of the SRA. For those antenna geometries with the cells of the MRA in the near field of the SRA, although the contributions from neighbor cells are still similar, the variation in the incidence angle of the field contributions from separated cells can be important. In this case the SRA is divided in groups of elements. The field contribution on every cell of the MRA is computed for each group of elements of the SRA and the incidence angle will be different for the contribution of each group impinges. This angle is considered for the computation of the reflection matrix of each pair SRA group and MRA cell.

The reflected coefficients on every cell of the MRA, are computed as many times as the number of groups in which the SRA is divided. The size of the groups is selected in such a way, that the far field conditions on every cell of the MRA are fulfilled for every group of the SRA. 

**Figure 1:**
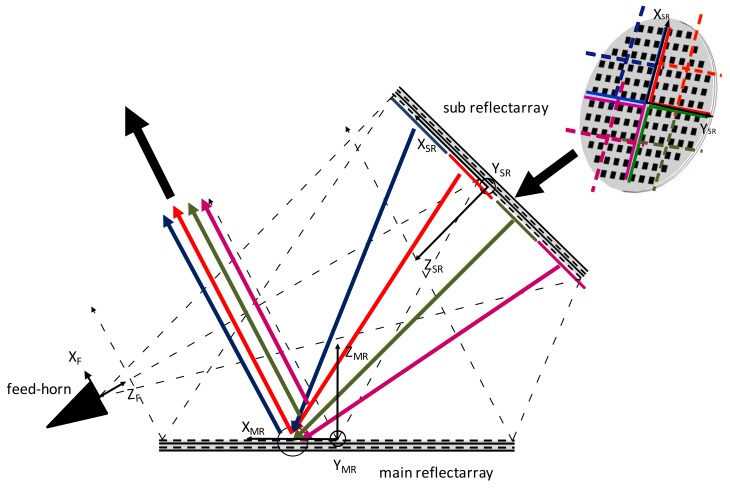
Scheme of the dual-reflectarray designed antenna with the sub-reflectarray divided in groups of elements.

All the field contributions of the elements belonging to the same group are added up before computing its corresponding reflected coefficient matrix. The incident angle is calculated assuming that each of those contributions starts in the center of its group. Equations (1) and (2) show the incident and reflected field on every (*m*,*n*) cell of the MRA due to one group of cells of the SRA:

(1) $$E_{inc\left(g\right)}^{X/Y}\left(m,n\right)=\sum_{r=1}^{Ngx}\sum_{s=1}^{Ngy}E_{inc\left(r,s\right)}^{X/Y}\left(m,n\right),$$

being Ngx and Ngy the number of elements for each group (g) in the *x* and *y* axis respectively. $E_{inc\left(g\right)}^{X/Y}\left(m,n\right)$ is the electric field impinging on the cell (*m*,*n*) of the MRA from the group of cells (g) of the SRA, according to Figure 1, for *X* or *Y* polarization. The tangential reflected field at each cell (m,n) of the MRA is:

(2) $$E_{ref}^{X/Y}\left(m,n\right)=\sum_{g=1}^{NG}R_{g}\left(m,n\right)\cdot E_{inc\left(g\right)}^{X/Y}\left(m,n\right),$$

where:

(3) $$R_{\left(g\right)}\left(m,n\right)=\left(\begin{array}{cc} {\rho_{\left(g\right)}^{m,n}}_{xx} & {\rho_{\left(g\right)}^{m,n}}_{yx}\\ {\rho_{\left(g\right)}^{m,n}}_{xy} & {\rho_{\left(g\right)}^{m,n}}_{yy} \end{array}\right).$$

$R_{\left(g\right)}\left(m,n\right)$ is the reflection matrix for the cell (*m*,*n*) of the MRA due to the group g of the SRA and NG is the number of groups. The components ${\rho_{\left(g\right)}^{m,n}}_{xx}$ and ${\rho_{\left(g\right)}^{m,n}}_{yx}$ are the direct and cross polarization reflection coefficients for an incident wave polarized in X_SR_ direction (without component in Y_SR_). On the other hand, ${\rho_{\left(g\right)}^{m,n}}_{yy}$ and ${\rho_{\left(g\right)}^{m,n}}_{xy}$ are the reflection coefficients for incident electric with Y_SR_ polarization.

The discussed analysis technique is a general approach; the two extreme cases correspond to a full-wave analysis and the fixed single angle analysis. In the first one, each group of elements contains only one element and in the second one, there is only one group of elements including all cells of the SRA. In the full-wave analysis, the incident angle of the electric field coming from each cell of the SRA impinging on each cell of the MRA, is taken into account. In the fix-angle case, there is only one angle of incidence to consider from all the field contributions from the SRA elements as it was considered in [24].

Finally, the 2D Inverse Discrete Transform computes the co-polar and cross-polar components of the radiation patterns by using an 2D-FFT based algorithm.

## 3. Design of a Contoured Beam Dual-Reflectarray Antenna

A demonstrator with limited size has been manufactured and tested to provide a contoured beam for European coverage. The antenna geometry main parameters are summarized in Table 1, they are similar to the antenna previously reported in [27]. The antenna geometry, including the relative positions of the feed, the SRA and the MRA remain equal to the antenna presented in [27]. The phase distribution on the SRA is also the same and emulates a hyperboloid to produce an equivalent parabolic reflector system with F/D=4. In this design the main difference lies on the phase distribution of the MRA that has been synthesized to provide a contoured beam in order to fulfill the requirements of a DBS mission.

A dual-linear polarized corrugated horn illuminates the SRA with the electric field polarized in *X*- and *Y*-axis of the feed coordinate system. The antenna is accommodated with the *Y*-axis of the MRA pointing to the North of the Earth, the V-polarized field is parallel to the *Y*-axis of the MRA. Using a dual circular horn, the antenna could operate in dual circular polarization since both linear polarizations are provided to the coverage. In the antenna geometry presented, the SRA is in the near field of the horn. A near field model of the feed-horn [29,30,31,32] has been used to make an accurate analysis, based on the spherical mode wave expansion of the pattern. The feed illuminates the SRA with a taper of −13.48 dB at the edges and the MRA is illuminated with −16.24 dB. The phase distribution on the SRA is computed using ray tracing and the phase shift in each cell is calculated as a function of the difference between the cell to the feed and the virtual focus of the antenna [27], as shown in Figure 1.

### 3.1. Phase Synthesis

**Figure 2:**
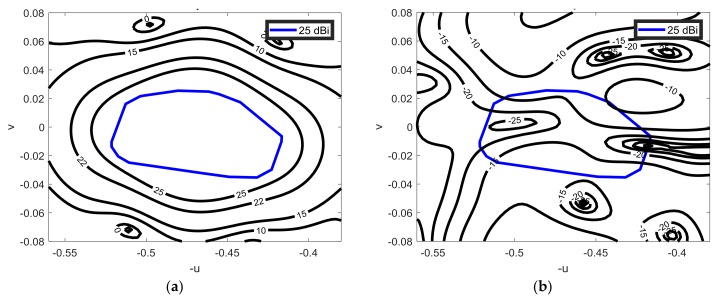
Synthesized pattern for vertical polarization at 13.75 GHz superimposed to the coverage mask: (**a**) co-polar component; (**b**) cross-polar component.

The phase synthesized provide an European coverage from a geostationary satellite located at 10° E longitude and 0° latitude. A minimum required gain of 25 dBi including beam pointing error in the enlarge region is defined. The phase distribution on the MRA is synthesized to provide the coverage defined by the blue line in Figure 2. 

The synthesis of the phase distribution on the MRA to produce a contoured beam is done using the technique based on the intersection approach algorithm reported in [32]. In the SRA, the phase-shift is calculated as a function of the difference between two distances: the distance between the virtual focus of the equivalent parabolic system and every cell of the SRA, and the distance from the feed-horn and every cell of the SRA. Figure 2 shows the superposition of the required mask for the European coverage, and the computed radiation pattern using the phase-shift distribution that fulfills the requirements. The radiation pattern fulfills the 25 dBi gain requirements, but it does not follow the gain contours of the mask accurately due to the limited size of the demonstrator.

### 3.2. Design of Main- and Sub-Reflectarrays

After computing the illumination and the phase distribution on both surfaces, next step consists on the design of both SRA and MRA. The dimensions of the patches are computed assuming local periodicity and the iterative process based on SD-MoM reported in [6]. The design has been carried out for dual linear polarization at 13.75 GHz. Table 2 shows the sandwich configuration where the same thickness and electric properties are considered for both reflectarray surfaces. The same sandwich structure with two layers of printed patches in a rectangular lattice is used for the SRA and MRA. The period of the regular layout for the MRA is 12 mm × 12 mm, it is arranged in 41 × 37 elements. For the SRA, the period is 10 mm × 10 mm arranged in a grid layout of 38 × 38 elements.

**Figure 3:**
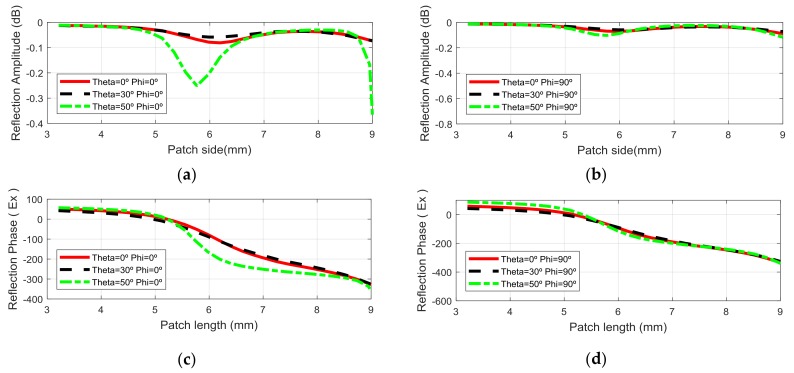
Reflection coefficient vs. the patch length in the first layer for the cell used in the MRA for: (**a**) Amplitude and; (**c**) Phase of EX field component at φ = 0° (Horizontal polarization) and; (**b**) Amplitude and (**d**) Phase of EY field component, φ = 90° (Vertical polarization) at 13.75 GHz.

Figure 3 shows the phase-shift as a function of the patch size at the frequency design (13.75 GHz) for 0°, 30° and 50° incident angle. The curves show a very linear variation over 400° on patches with a lateral size between 4 mm and 9 mm on the first layer, for up to 50° incident angles for both linear polarizations. 

The average losses for the patch sandwich are lower than 0.1 dB. In Figure 3c, there are phase variations of up to 80° in the MRA and 30° in the SRA for 50° incidence angles. For 9 mm patch lengths and 50° incident angles, the losses increase drastically due to the resonance, these patches are closer to the edge of the reflectarray where the illumination is 10 dB lower than in the center, their effect is negligible.

## 4. Comparison between Simulations and Experimental Results

For the validation of the process, the designed dual-reflectarray antenna has been analyzed, manufactured and then tested in an anechoic chamber. The analysis has been carried out with different degrees of accuracy to demonstrate the efficiency of the analysis technique. In the first analysis, groups of one element in the SRA are considered, what it has been previously mentioned as full-wave analysis. 

Then, the approximation of the fix incident angle is used in the analysis where only one group of elements in the SRA is considered. Then the antenna is analyzed when dividing the SRA in a progressive higher number of groups, increasing the accuracy in the radiation pattern prediction, until getting closer to the results obtained with the full wave analysis, ensuring the convergence of the analysis method. To compute the optimum number of groups in the SRA that makes the results to converge to the full-wave case analysis, with a reasonable computational time. The maximum group size in the SRA is calculated to fulfill the far field conditions with every cell of the MRA. Considering the minimum distance between the sub and the main reflectarray, 236 mm, the diameter of one group in the sub must be less than 50 mm to fulfill far-field conditions and between 50 mm and 140 mm to be in the Fresnel zone. With a period of 10 mm in the SRA, groups of 5 × 5 elements would be in the far field of the cells in the MRA. The SRA has 38 × 38 elements, when making groups of 13 × 13 elements, 9 groups are considered in the analysis, when making groups of 8 × 8 elements, 25 groups are considered in the analysis. Still, with 25 groups, the elements of the MRA closer to the SRA are in the Fresnel Zone, but most of the elements in the MRA are in the far field of the 25 groups of the SRA.

**Figure 4:**
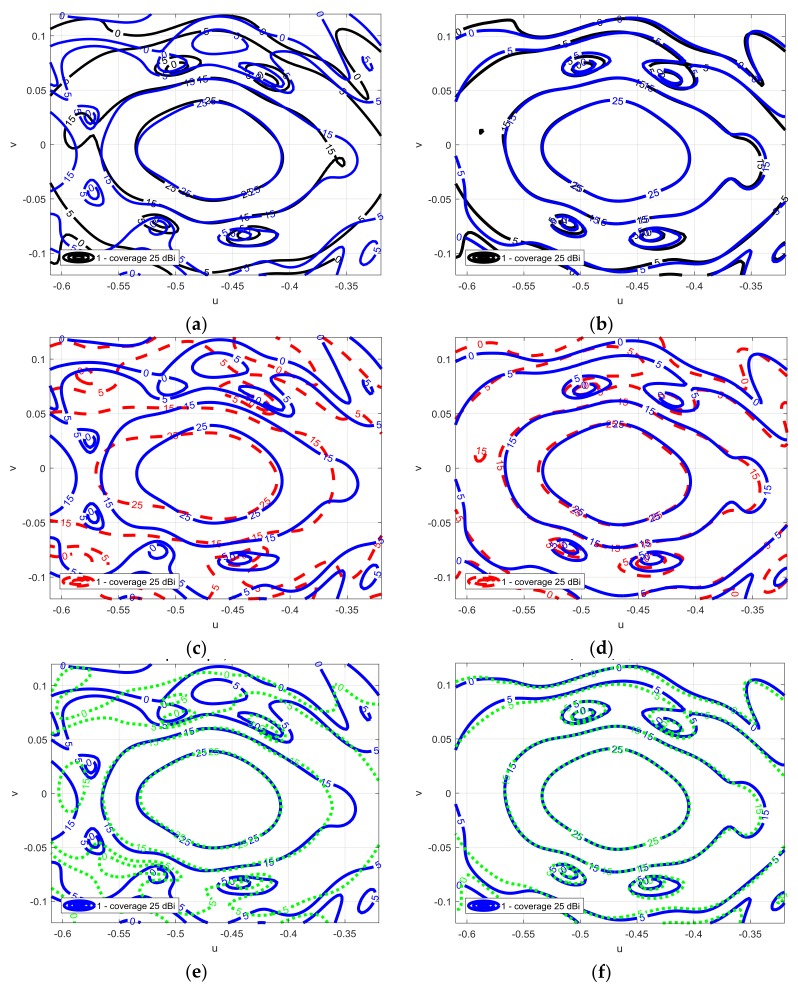
(**a**) Contour plots of the copolar components of the radiation pattern full wave analysis (solid blue line (-)) and approximate case (dashed black line (--)) vertical polarization; (**b**) for horizontal polarization. (**c**) Contour plots of the copolar components of the radiation pattern simulated for the full wave analysis (solid blue line (-)) and the more general approach dividing the SRA in 9 (dashed dot red line (-)) for vertical polarization and (**d**) for horizontal polarization. (**e**) Contour plots of the copolar components of the radiation pattern simulated for the full wave analysis (solid blue line (-)) and the more general approach dividing the SRA in 25 (dashed green line (:)) for vertical polarization and (**f**) for horizontal polarization.

Figure 4 shows the copolar component of the radiation pattern for several cases: for the full-wave analysis (blue line), when the SRA is divided into one (black line), nine (red line), 25 (green line) groups, for both linear polarizations. When the SRA is divided into nine groups, a good agreement in the main beam is achieved, when it is divided in 25 groups, the green line converges to the full-wave case. For vertical polarization, the fix-angle approach is good since the reflectarray elements are not very sensitive to the incidence angle for 9 mm patch dimensions. Performing an analysis on an antenna, like the one presented in the paper requires a computational time of 100 h in the case of the full-wave analysis and 3 min using the approximated method, using the same computer set-up.

**Figure 5:**
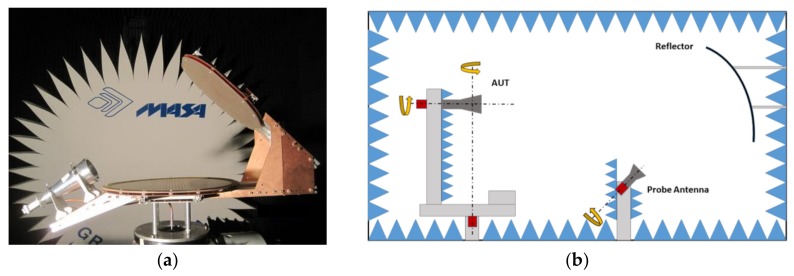
(**a**) Dual-reflectarray antenna for contoured beam in the compact antenna test rage (anechoic chamber) (**b**) Sketch of the CATR measurement system.

As shown in Figure 5, the prototype of the antenna has been measured in the Compact Antenna Test Range (CATR) of Universidad Politécnica de Madrid in an anechoic chamber. In this measurement system, the probe is placed at the focus of a parabolic reflector so that, the reflector transforms the spherical wave coming from the probe to a plane wave that illuminates the Antenna Under Test (AUT). This system permits to measure the antenna in far field conditions in a smaller room that a conventional spherical range system. The AUT is placed in the quite zone of the test range which is the volume where the plane wave conditions are fulfilled. The AUT can be rotated along two axes (roll and azimuth) so the entire sphere can be measured by the probe, while the RX antenna can be rotated along its axis so the co and crosspolar patterns can be measured.

**Figure 6:**
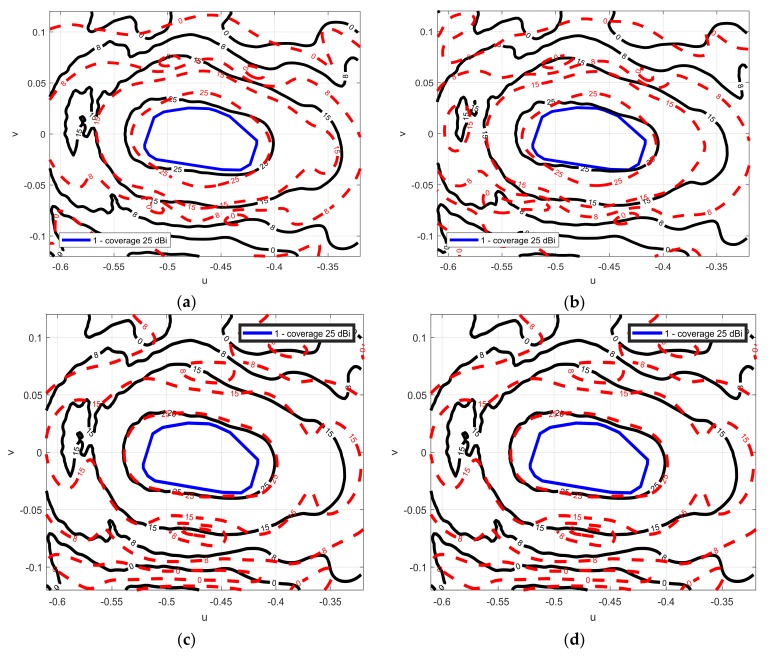
(**a**) Contour plots of the simulated copolar components of the radiation pattern (dashed red line (--)), measurements (solid black line (-)) and the mask (thicker solid blue line) for vertical polarization at 13.75 GHz; (**b**) for horizontal polarization at 13.75 GHz; (**c**) The simulations are repeated accounting for the phase errors (red line) and compared with measurements for vertical polarization; (**d**) for horizontal polarization.

The measurements and the simulated radiation pattern fulfill the requirements for the three measured frequencies (12.975, 13.75 and 14.25 GHz). Good agreement between simulations (25 groups in the SRA) and measurements at the central frequency is achieved. The small misalignment between the layers of the printed patches on the MRA produces small mismatching between simulations and measurements. Those misalignments produced up to 30° phase errors in some areas of the reflectarray where the incident angles are high. When considering theses phase errors in the analysis of the antenna and making a new comparison with the measurements, much better agreement was achieved as shown in Figure 6c,d. 

**Figure 7:**
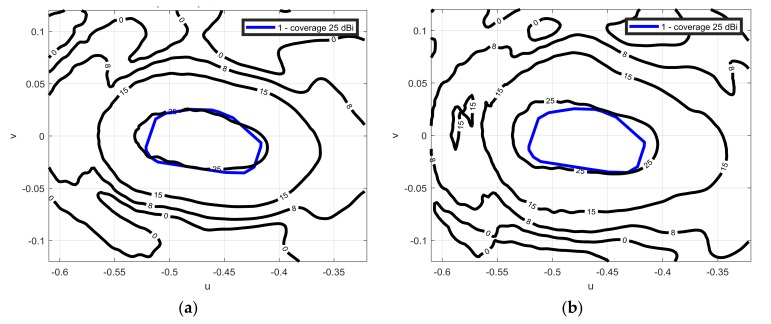
Measured contoured patterns in dBi (solid black line) superimposed to the coverage mask (thicker solid blue line) for vertical polarizations: (**a**) at 12.975 GHz; (**b**) at 14.25 GHz.

**Figure 8:**
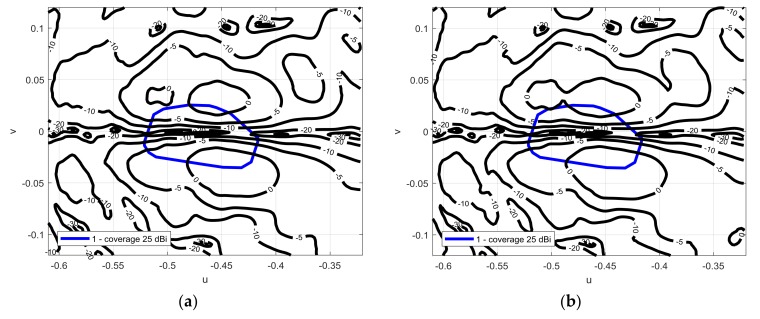
(**a**) Measured contoured plots of the cross-polar components of the radiation pattern (solid black line) superimposed to the coverage mask (thicker solid blue line) for vertical polarization at 13.75 GHz; (**b**) for horizontal polarization at 13.75 GHz.

The antenna was measured in the frequency range (12.975–14.25 GHz), as Figure 7 points out, a very stable performance in the required frequency band was achieved. The cross polar discrimination is larger than 25 dB, Figure 8 shows the crosspolar component of the radiation pattern for both linear polarizations at 13.75 GHz.

## 5. Conclusions

This article shows the validation of a general and accurate analysis technique for dual-reflectarray antennas. When computing the incident and reflected field on every cell of the MRA with this technique, the subreflectarray (SRA) is divided in groups of elements. The contribution of each group is considered for every element of the main reflectarray (MRA) that is analyzed. An increase on the number of groups derives in an increase of the computation time but also an improvement of the accuracy. A limited size demonstrator was designed, manufactured and tested to validate the analysis technique. The MRA size is 450 mm × 500 mm and it provides a contoured beam for DBS European coverage with dual linear polarization. The cross-polarization discrimination is higher than 25 dB and remains stable within the frequency range: from 12.975 GHz to 14.25 GHz. Although, the comparison between the simulations and the measurements shows a good agreement, there are slight differences in the shape of the patterns due to manufacturing tolerance errors.

This paper demonstrated the capability of dual-reflectarray antennas to provide contoured beams with low levels of cross-polarization in Ku-band. A similar configuration could be used for multi-spot antennas in Ka-band, since the simultaneous phase adjustment in two surfaces can be used for multi focal designs.

## Figures and Tables

**Table 1:** Geometrical parameters of the dual-reflectarray antenna.

**Main Reflectarray (MRA)**	
Main-Reflectarray size	492 mm × 444 mm
Rows × Columns	41× 37 elements
Period	12 mm × 12 mm
**Sub-Reflectarray (SRA)**	
Center of the coordinate system	(−217, 0, 370) mm
Direction cosines matrix (relates MRA and SRA Coordinate Systems)	$\left[\begin{array}{ccc} 0.715 & 0 & 0.698\\ 0 & -1 & 0\\ 0.698 & 0 & -0.715 \end{array}\right]$
Sub-Reflectarray size Rows × Columns	380 mm × 380 mm 38 × 38 elements
**Feed-Horn**	
Coordinates of phase-center	(193.73, 0, 635.54) mm
Intersection of feed axis and SRA	(15, 0, 0) mm
Coordinates of phase-center	(193.73, 0, 635.54) mm

**Table 2:** Lay-up of the sandwich and radio-electric characteristics of the materials.

Dielectric/Metallization	ε_r_	Tag δ	Thickness (mm)
Copper (Printed Patch Layer 2)	-	-	0.05
(SRA) Cuclad 217LX/(MRA)Diclad880B	2.17	0.0009	1.524
(SRA/MRA)Cuclad6250 (Thermoplastic bonding film)	2.32	0.0013	0.038
Copper (Printed Patch Layer 1)	-	-	0.05
(SRA) Cuclad 217LX/(MRA)Diclad880B	2.17	0.0009	1.524
Copper (Ground Plane)	-	-	0.05
